# Improving Dust Aerosol Optical Depth (DAOD) Retrieval from the GEOKOMPSAT-2A (GK-2A) Satellite for Daytime and Nighttime Monitoring

**DOI:** 10.3390/s24051490

**Published:** 2024-02-25

**Authors:** Soi Ahn, Hyeon-Su Kim, Jae-Young Byon, Hancheol Lim

**Affiliations:** 1 Environmental Satellite Center (ESC), National Institute of Environmental Research (NIER), 42, Hwangyeong-ro, Seo-gu, Incheon 22689, Republic of Korea; jjahn@korea.kr; 2 National Meteorological Satellite Center (NMSC), Korea Meteorological Administration (KMA), Jincheon-gun 27803, Republic of Korea; atmos1305@korea.kr (H.-S.K.); hclim09@korea.kr (H.L.)

**Keywords:** dust aerosol optical depth (DAOD), GK-2A, thermal infrared, dust, cumulative distribution function (CDF), aerosol robotic network (AERONET)

## Abstract

The Advanced Meteorological Image (AMI) onboard GEOKOMPSAT 2A (GK-2A) enables the retrieval of dust aerosol optical depth (DAOD) from geostationary satellites using infrared (IR) channels. IR observations allow the retrieval of DAOD and the dust layer altitude (24 h) over surface properties, particularly over deserts. In this study, dust events in northeast Asia from 2020 to 2021 were investigated using five GK-2A thermal IR bands (8.7, 10.5, 11.4, 12.3, and 13.3 μm). For the dust cloud, the brightness temperature differences (BTDs) of 10.5 and 12.3 μm were consistently negative, while the BTD of 8.7 and 10.5 μm varied based on the dust intensity. This study exploited these optical properties to develop a physical approach for DAOD lookup tables (LUTs) using IR channels to retrieve the DAOD. To this end, the characteristics of thermal radiation transfer were simulated using the forward model; dust aerosols were explained by BTD (10.5, 12.3 μm)—an intrinsic characteristic of dust aerosol. The DAOD and dust properties were gained from a brightness temperature (BT) of 10.5 μm and BTD of 10.5, 12.3 μm. Additionally, the cumulative distribution function (CDF) was employed to strengthen the continuity of 24-h DAOD. The CDF was applied to the algorithm by calculating the conversion value coefficient for the DAOD error correction of the IR, with daytime visible aerosol optical depth as the true value. The results show that the DAOD product can be successfully applied during the daytime and nighttime to continuously monitor the flow of yellow dust from the GK-2A satellite in northeast Asia. In particular, the validation results for IR DAOD were similar to the active satellite product (CALIPSO/CALIOP) results, which exhibited a tendency similar to that for IR DAOD at night.

## 1. Introduction

To understand the current and future environmental challenges, we must characterize the role of atmospheric aerosols in various meteorological processes across different environments, impacting climate, weather, and air quality. Aerosol particles markedly impact visibility [1,2], dimming [3], precipitation [4], air quality [5], and human health, including blood circulation [6].

Mineral dust aerosols are a crucial atmospheric component, causing variabilities in interactions with clouds by impacting their optical properties. In this way, dust aerosols influence the radiative budget and global climate change by contributing to abnormal weather. 

In northeast Asia, the wind-driven movement of aerosols relies on meteorological conditions and affects the air quality. In particular, dust storms occur frequently during the springtime, causing poor air quality in northeast Asia. In recent years, the frequency of yellow dust events has increased in the winter and fall as they have become drier due to climate change. Therefore, it is an essential phenomenon for understanding Earth’s climate circulation system. 

Satellite monitoring is an important strategy for scrutinizing the properties of the different aerosol flow types: mineral dust, fine particles, and volcanic ash. The aerosol optical depth (AOD) retrieved from satellites is the best indirect measurement for atmospheric environmental research modeling studies. Accordingly, increased research attention has been focused on reducing associated errors and, thus, improving the accuracy of AOD [7]. Many ground-based remote-sensing aerosol networks, including the aerosol robotic network (AERONET), have been established globally. Additionally, basic optical properties are derived from numerous Earth observation satellites, such as Geosynchronous Earth Orbit (GEO) satellites, including the Advanced Baseline Imager (ABI), Advanced Himawari Imager (AHI), Advanced Meteorological Imager (AMI), and Geostationary Ocean Color Imager (GOCI). Furthermore, aerosol retrieval algorithms have been developed for Low Earth Orbit (LEO) satellite instruments, such as the Advanced Very High-Resolution Radiometer (AVHRR) [8,9], Ozone Monitoring Instrument (OMI) [10], Multiangle Imaging Spectro-Radiometer (MISR) [11], Moderate Resolution Imaging Spectrometer (MODIS) [12], Visible Infrared Imaging Radiometer Suite (VIIRS) [13,14], and Cloud and Aerosol Imager (CAI) [15]. 

Previous studies on aerosol detection via satellite have performed measurements using the visible channel. Meanwhile, GEO and LEO satellites have various visible and near-IR channels, enabling corrected reflectivity and aerosol property retrieval with high accuracy. However, aerosol observations using the visible channel are limited to the daytime. Accordingly, we sought to study dust aerosol in the nighttime IR spectral domain using the Atmospheric Infrared Sounder (AIRS) or Infrared Atmospheric Sounding Interferometer (IASI) [16,17]. However, to achieve this, IR spectral observations must complement solar measurements. First, the terrestrial radiative forcing can be determined based on the effect of dust on IR radiation [18]. Excluding bright surfaces, such as snow and deserts, it is important to accurately observe the absorption of longwave radiation emitted from the Earth’s surface [19]. Second, the sensitivity of DOAD is considerably impacted by the size of aerosol particles. For example, coarse modes, such as mineral aerosols, are observed in the IR domain. In contrast, pollution is derived in the visible and IR domains, making it difficult to discriminate between them. Third, IR observations can be observed at night, allowing the flow of dust to be monitored, which has proven practical in acquiring aerosols regardless of surface characteristics [20,21,22,23].

The objectives of this study were to develop an advanced DAOD algorithm to identify dust-storm outbreaks and their spatial extent using IR channels from Geo-Kompsat 2A (GK-2A). Since only IR channels were used, the algorithm was applicable to daytime and nighttime conditions. The DAOD of the IR domain was derived using a physical method based on offline calculated look-up tables (LUTs). Furthermore, to increase the accuracy of the DAOD, the CDF method was applied to analyze its qualitative and quantitative performances.

## 2. Materials and Methods

### 2.1. Materials

#### 2.1.1. Satellite Observations: GEOKOMPSAT-2A (GK-2A)

The GK-2A of the Korean Meteorological Administration (KMA) has been performing a meteorological satellite mission since 5 December 2018, and is currently operating well. The GK-2A life is 10 years; its capability with multi-band and high temporal and spatial resolution was verified during the 8 months in-orbit test period. Currently, GK-2A AMI scans the Earth’s full disk every 10 min and the Korean Peninsula every 2 min over using high spatiotemporal resolution (0.5–2 km). Similar to the GOES-16 (National Aeronautics and Space Administration, NASA, Washington, DC, USA) and Himawari-8 (Japan Aerospace Exploration Agency, JAXA, Tokyo, Japan), GK-2A has 16 channels: visual channels (0.47, 0.51, 0.64, and 0.86 μm), near-IR channels (1.38 and 1.61 μm), and IR channels (3.8, 6.3, 6.9, 7.3, 8.7, 9.6, 10.5, 11.2, 12.3, and 13.3 μm) (Table 1). This satellite greatly improves the accuracy of precision weather and prognosticates extreme weather over the Korean Peninsula and the Asia-Pacific region. GK-2A AMI diversifies channels and provides RGB color images using compositing techniques. This allows real-time monitoring of various weather phenomena. Moreover, it can be used for unpredictable and dangerous weather through continuous observation.

#### 2.1.2. AERONET

The Aerosol Robotic Network (AERONET) is an international ground-based aerosol remote sensing network [23] (https://aeronet.gsfc.nasa.gov/ accessed on 1 January 2020). It provides representative data to validate aerosol product data from the satellite. The AERONET AOD uncertainty at Level 2 (version 2) is approximately 0.01 to 0.02, which can be considered a true value to validate AMI AOD over land and ocean. In this study, AERONET data was used to validate the accuracy of DAOD by performing a spatiotemporal collocation between AMI AOD 11.4 μm and AERONET observations. AERONET observations within a 10-min span were averaged to perform ground-measured aerosol loading; averaged AMI 11.4 μm AODs within a 10-km radius of one AERONET site were used to represent satellite aerosol movement. 

#### 2.1.3. Suomi-NPP/VIIRS

The Visible Infrared Imaging Radiometer Suite (VIIRS) instrument aboard the Suomi National Polar-Orbiting Partnership (S-NPP) satellite was launched in October 2011 and was extend the performance of MODIS. VIIRS has various channels (visible, short, IR spectral), providing numerous products with precise accuracy [24]. In particular, VIIRS aerosol data (VIIRS AOD550s at Intermediate Product level) were computed in pixel mode with a spatial resolution of 0.75 km. Hence, unlike MODIS, it can validate the accuracy of products with high spatial resolution. The VIIRS product was downloaded from the National Oceanic and Atmospheric Administration’s (NOAA)’s Comprehensive Large Array-Data Stewardship System (CLASS) (https://www.ncei.noaa.gov, accessed on 1 January 2020). The data have undergone continuous evaluations with uncertainty ranges of [∆τ=−0.470τ−0.01 (lower bound), −0.0058τ+0.09 (upper bound)] over land and [∆τ=−0.238τ+0.01  (lower bound), 0.194τ+0.048 (upper bound)] over ocean [13]. The VIIRS aerosol product was used to verify the accuracy of the DAOD developed in this study for the yellow sand period in 2020. 

#### 2.1.4. CALIPSO/CALIOP

The Cloud-Aerosol Lidar with Orthogonal Polarization (CALIOP), the main instrument of the Cloud-Aerosol Lidar and Infrared Pathfinder Satellite Observation (CALIPSO) spacecraft, is a major sensor that studies interactions between aerosols, clouds, and vertical profiles. Particularly, CALIPSO derives profiles of attenuated backscatter at 532 and 1064 nm and polarized backscatter at 532 nm. This study used an active sensor capable of retrieving information about aerosols and clouds during the daytime and nighttime. Also, CALIPSO has high horizontal and vertical resolutions of 333 m and 30–60 m, respectively, facilitating the evaluation of aerosol (e.g., aerosol type, extinction profile, height, optical depth), and cloud properties [25,26]. CALIPSO has three products levels that use raw signals to distinguish clouds and aerosols (L1), reclassifies the types (L2), and provides specific variables, such as extinction coefficients, AOD (L3). In this study, we used L3 data to validate DAOD at night when yellow dust occurred. 

#### 2.1.5. Dust Storm Events in 2020–2021

In this study, the dust storm events that occurred between 2020 and 2021 in Northeast Asia were the study objects as GK-2A satellite data were available for 24 h for the visible and IR channels of the retrieved DAOD. Therefore, the National Meteorological Satellite Center (NMSC) of KMA provided information on the optical depth monitoring of yellow dust while simultaneously identifying the flow of dust storms and selecting cases that affect the Korean Peninsula for use in forecasting (Table 2). This was applied to the dust event that occurred in the spring of 2021 using training data based on the dust storm in 2020. 

### 2.2. Methods

#### 2.2.1. Look-Up-Table-Based Physical Retrieval Algorithm

**Figure 1:**
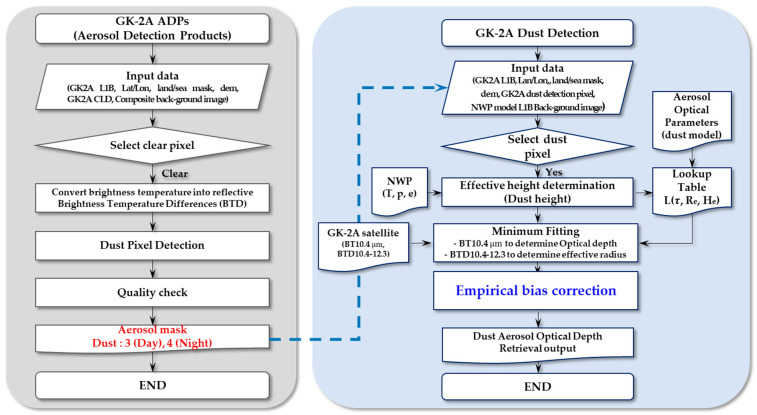
Flowchart of the Dust Aerosol Optical Depth (DAOD) product algorithm used by GK-2A/AMI.

The DAOD was retrieved during the day and night utilizing a GK-2A IR channel. A flowchart of the DAOD automated algorithm is shown in Figure 1. 

##### GK-2A Dust Pixel Retrievals

The GK-2A AMI Aerosol Detection Products (ADPS) algorithm can monitor the aerosol category (e.g., dust, haze, and volcanic ash) by fusing the multiple channels [27,28]. These characterizations and physical principles were used to determine the threshold values of the dust algorithm. A GK-2A ADPS algorithm based on the BTD method was developed to retrieve aerosol types to reflect daytime, nighttime land, and ocean characteristics differently. This study focused on dust pixels using thermal IR (TIR) channels. This method is widely used due to its advantage of monitoring dust at night and over bright surfaces, such as deserts. Assuming that the temperature most similar to the surface temperature was a clear pixel, the boundary value of ${BT}_{10.5}$ was used as the most basic value [29]. During the day, ${BT}_{10.5}$ decreases in the presence of dust due to the maximum dust absorption of 10.5 μm [30] and peaks in bright surfaces (Equation (1)).

(1) $${BT}_{10.5}>243.0\;K$$

The boundary value of this dust mask relies on the spatial and thermal distinctions between aerosols and clouds; the depression of the brightness temperature difference (BTD) between ${BT}_{10.5}$ and ${BT}_{12.3}$ (${BTD}_{10.5-12.3}$, Equation (2)) can be exhibited by the dust plumes [30,31,32,33,34]. Using the characteristic of preferentially absorbing shorter wavelengths, cloud components with silicate particles can be discriminated using various IR bands [35]. Silicate particles cause a negative BTD between ${BT}_{10.5}$ and ${BT}_{12.3}$ for the dust region, with the longer wavelength channel recording a higher brightness temperature [35]. ${BT}_{8.7}$ is not as affected by the presence of dust but is much lower than ${BT}_{10.5}$ under unspoiled sky conditions. Therefore, ${BT}_{8.7}$ and ${BT}_{10.5}$ (${BTD}_{8.7-10.5}$, Equation (3)) are larger for dust than for the ground and clouds [30,36,37,38,39]. To improve the accuracy of dust detection, 11.2 μm and 12.3 μm (${BTD}_{11.2-12.3}$, Equation (4)) have similar spectral characteristics, however, elicit strong signals for weak or low-altitude dust [40]. The brightness temperature ratio (BTR) between the two wavelength channels was built to more precisely discriminate between the dust and surface properties [41]. Typically, the ratios of ${BT}_{10.5}$ and ${BT}_{12.3}$ (${BTR}_{10.5-12.3}$) values are lower than over the dust region; therefore, in the algorithm, the boundary value was applied to increase the accuracy of dust detection (Equation (5)). Furthermore, the BTR value was applied by changing the channel values ${BT}_{10.5}$ and ${BT}_{8.7}$ (${BTR}_{10.5-8.7},$ Equation (6)). The BTD-BBTD (background) test uses 30-day background image data and, thus, corrects the water vapor (Equation (7)). D*-parameter (Equation (8)) is computed by combining the dust absorption rate difference of 8.7, 10.5, and 12.3 μm and the coefficient based on the empirical equation and is a representative variable that can identify yellow dust. This parameter was created by Hansell to detect nighttime dust since many silicate minerals with strong bands often absorb better at 8.7 μm than 10.5 μm, leading to a negative ${BTD}_{8.7-10.5}$. Therefore, a parameter is often designed such that a value >1 indicates dust and a value < 1 indicates clouds [42]. 

On land, Equations (2)–(8) were applied:

(2) $${BT}_{10.5}-{BT}_{12.3}\;\leq 0.1\;$$

(3) $${BT}_{10.5}-{BT}_{8.7}<-0.8\;$$

(4) $${BT}_{11.2}-{BT}_{10.5}\;\geq 0.5$$

(5) $$Ratio\left[\frac{{BT}_{10.5}}{{BT}_{12.3}}\right]\leq 0.1$$

(6) $$Ratio\left[\frac{{BT}_{10.5}}{{BT}_{8.7}}\right]\leq 0.1$$

(7) $$\left({BTD}_{10.5-12.3}\right)-\left(B{BTD}_{10.5-12.3}\right)$$

(8) $$D^{*}=exp\left\{\left[\left({BTD}_{10.5-12.3}\right)-C\right]/\left[\left({BTD}_{8.7-10.5}\right)-E\right]\right\}\;$$

where the offsets *C* and *E* were set to be −0.5 and 15.

In the ocean, Equations (9)–(15) were applied:

(9) $${BT}_{10.5}-{BT}_{12.3}\;\leq 0.1\;$$

(10) $${BT}_{10.5}-{BT}_{8.7}<-0.8\;$$

(11) $${BT}_{11.2}-{BT}_{10.5}\;\geq 0.5$$

(12) $$Ratio\left[\frac{{BT}_{10.5}}{{BT}_{12.3}}\right]\leq 0.1$$

(13) $$Ratio\left[\frac{{BT}_{10.5}}{{BT}_{8.7}}\right]\leq 0.1$$

(14) $$\left({BTD}_{10.5-12.3}\right)-\left(B{BTD}_{10.5-12.3}\right)$$

(15) $$D^{*}=exp\left\{\left[\left({BTD}_{10.5-12.3}\right)-C\right]/\left[\left({BTD}_{8.7-10.5}\right)-E\right]\right\}\;$$

In this study, the DAOD using IR channels was calculated using only the dust pixels identified through the threshold test. 

##### Forward Simulation

To defined the BT and BTD (10.5 and 12.3 μm) characteristics of dust, an appropriate aerosol model was selected. The dust aerosol model must be developed considering the complex refractive index, particle size distribution, and particle shape. The complex refractive index (RI) is a parameter that defines the interaction between electromagnetic radiation and matter [43]. The real part indicates the scattering properties and the imaginary part declares the absorption properties of the dust aerosol (Equation (16)).

(16) $$Refractive\;Index\left(RI,\lambda \right)=n\left(\lambda \right)-ik\left(\lambda \right)$$

where λ = wavelength, *n* = real part, and *k* = imagery part [44,45].

The particle size distribution is expressed as a lognormal distribution. During yellow dust, large particles become principal, and the lognormal distribution was selected per Equation (17):

(17) $$\frac{dv}{dln\;r}=\frac{C_{fine}}{\sqrt{2\pi S_{fine}}}\exp\left[-\frac{{(\ln r-\ln r_{fine})}^{2}}{{2\left(S_{fine}\right)}^{2}}\right]+\frac{C_{coarse}}{\sqrt{2\pi S_{coarse}}}\exp\left[-\frac{{(\ln r-\ln r_{coarse})}^{2}}{{2\left(S_{coarse}\right)}^{2}}\right]$$

where *dv/dlnr* is the particle number size distribution; $C_{fine}$ and $C_{coarse}$ are the number of particles per cross-section of the atmospheric column ($m^{-2})$; *r* is the particle radius; $r_{fine}$ and $r_{coarse}$ are the modal radii; and $S_{fine}\;$ and $S_{coarse}$ are the standard deviations of $lnr_{fine}$ and $lnr_{coarse}$, respectively.

The shape of aerosol particles is difficult to define owing to their irregular shape and non-uniform size; therefore, the particles were assumed to be spherical in this study. The phase function can be expressed by Equation (18):

(18) $$P\left(\lambda ,\theta \right)=\int_{r=0}^{r=\infty }\pi r^{2}F\left(\lambda ,r,RI,\theta \right)\frac{dN}{dlnr}dlnr$$

where *F* is the top of the atmosphere (TOA) radiative forcing in W/m^2^; *N* is the particle number density; and *r* is the particle radius. 

DISORT is a discrete ordinate radiative transfer program for sorting the radiative transfer equation [46]. The IR radiative transfer of the dust layer was simulated using previously calculated parameters (i.e., complex refractive index, particle size distribution, shape) and the values were derived for the single scattering albedo ($\omega_{0}$), asymmetry factor (g), and extinction coefficient ratio (k) based on Equations (19)–(21):

(19) $$Extinction\;Coefficient:\sigma_{ext}\left(\lambda \right)=\sigma_{sca}\left(\lambda \right)+\sigma_{abs}\left(\lambda \right)$$

(20) $$Single\;Scattering\;Albedo:\omega_{0}\left(\lambda \right)=\frac{\sigma_{sca}\left(\lambda \right)}{\sigma_{ext}\left(\lambda \right)}$$

(21) $$Asymmetry\;Factor\;g\left(\lambda \right)=\frac{\int cos\theta P\left(\lambda ,\theta \right)dcos(\theta )}{\int \left(\lambda ,\theta \right)dcos(\theta )}$$

**Figure 2:**
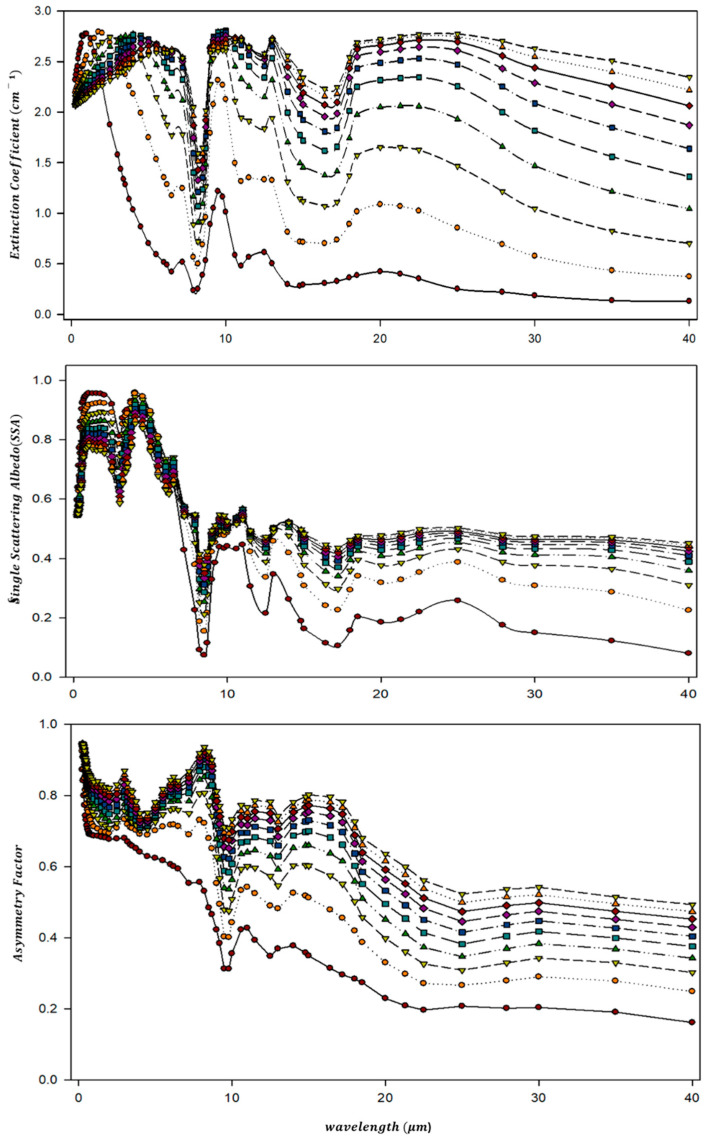
Simulation model results based on infrared channel characteristics by DISORT (1 model (red circle), 2 model (orange circle), 3 model (yellow inverted triangle), 4 model (green triangle), 5 model (sky blue square), 6 model (blue square), 7 model (purple rhombus), 8 model (burgundy rhombus), 9 model (orange triangle), 10 model (yellow green inverted triangle)).

These calculated parameters together with a given optical depth of the dust layer, serve as input for the radiation transfer model, DISORT, to simulate IR wavelength observations (Figure 2).

##### DAOD Look-Up Table

The forward radiative transfer model in IR channels is expressed by Equation (22):

(22) $$I_{TOA}=\epsilon_{c}\;I_{ac}+T_{ac}\epsilon_{c}\;B\left(T_{c}\right)+I_{clr}\left(1-\epsilon_{c}\right)\;$$

where $I_{TOA}$ is the satellite-received radiance; $I_{ac}$ is the radiance contribution from the region above the dust cloud; $I_{clr}$ is the clear-sky radiance; $T_{ac}$ represents the above-dust cloud transmission; $T_{c}$ is the effective top temperature of dust layer; *B* operator is the Planck function; and ($\epsilon_{c}$) is the emissivity at the top of the dust layer. 

The cloud is defined by its cloud top temperature $T_{c}$ and its emissivity $\epsilon_{c}$. In Equation (22), the transmission from the dust layer, dust cloud top temperature, and Planck function are related to dust extinction at IR wavelengths. By removing other terms, the DAOD can be derived analytically. However, surface and dust cloud emissivity are relatively ambiguous, and the dust optical values were retrieved using LUTs calculated by radiative transfer [47]. The retrieval strategy was based on a physical approach that relied on the use of LUTs for the simulated DAOD (Table 3). To derive accurate DAOD, the measured top-of-atmosphere (TOA) radiance is a crucial to computing the LUT using an appropriate aerosol dust model. TOA radiance was calculated using the Santa Barbara discrete ordinate radiative transfer (SBDART) (http://libradtran.org, accessed on 1 January 2018) [48].

##### Estimation of the Effective Dust Height

This study assumed a method using the GOES-R volcanic ash height [49]. This method was assumed to be similar to inferring the height of volcanic ash by searching a temperature close to the height when volcanic ash moves. The retrieved $T_{eff}$ was used to approximate the dust height and find the closest matching temperature point; the linear interpolation weights and points were decided by finding $T_{eff}$ within the Numerical Weather Prediction (NWP) temperature profile. In this study, the vertical NWP profiles utilized of dust retrieval were assigned a temperature that reflected the levels between the surface and the model tropopause height (~10 km). Subsequently, the dust height was computed using the interpolation method (Equation (23)):

(23) $$H_{Dust}\left(height\right)=H_{1}+\frac{T_{eff}-T_{1}}{T_{2}-T_{1}}\left(H_{2}-H_{1}\right)\;$$

where $H_{Dust}$ is the dust height; $T_{1}$ and $T_{2}$ are the temperatures within the profile that bound $T_{eff}$, with $T_{1}$ as the temperature at the highest bounding level; and $H_{1}$ and $H_{2}$ are the dust heights of the bounding temperatures corresponding to $T_{1}$ and $T_{2}$, respectively [49]. 

**Figure 3:**
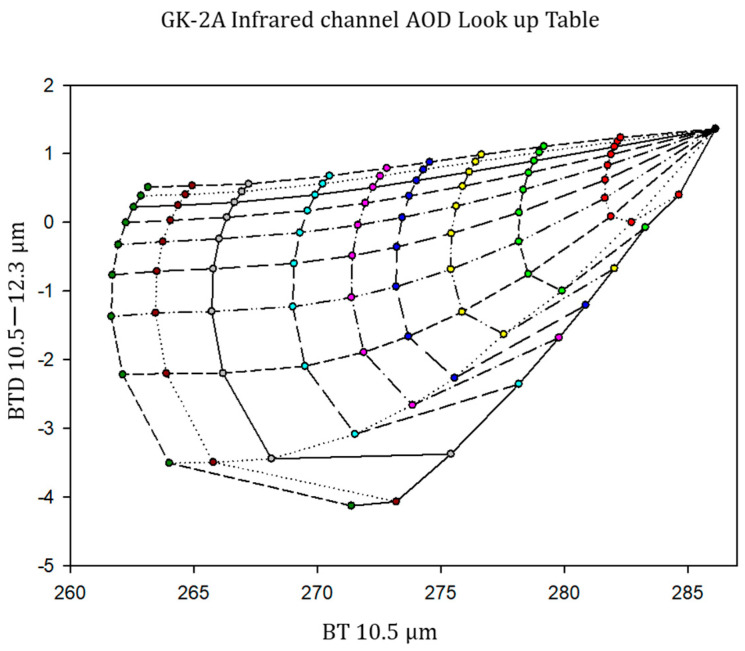
Example of the look-up table calculated according to the variation in optical depth and effective radii of dust particles.

Based on forward simulations, it was deemed appropriate to develop the temperature-difference model according to height ($H_{Dust}$). The temperature difference model included ${BT}_{10.5}$ and ${BTD}_{10.5-12.3}$. The ${BT}_{10.5}$ and ${BTD}_{10.5-12.3}$ values calculated from the LUTs (simulated BT) were compared with the ${BT}_{10.5}$ and ${BTD}_{10.5-12.3}$ values calculated from the satellite (observed BT). ${BT}_{10.5}$ is highly correlated with dust optical depth and ${BTD}_{10.5-12.3}$ nearby corresponds to particle volume [43]. The value with the minimum root-mean-square deviation (RMSD) was determined through comparison (Equation (24)); the optical depth and particle effective radii of the dust were obtained at the same time. Therefore, the accuracy of $T_{ac}$ and $T_{c}$ determines the reliability of the retrieved results (Figure 3).

(24) $$Min\;RMSD=\frac{1}{N}\sqrt{\frac{{\left({BT}_{10.5\left(calc\right)}-{BT}_{10.5\left(obs\right)}\right)}^{2}}{{BT}_{10.5\left(obs\right)}}}+\frac{1}{N}\sqrt{\frac{{\left({BTD}_{10.5-12.3\left(calc\right)}-{BTD}_{10.5-12.3\left(obs\right)}\right)}^{2}}{{BTD}_{10.5-12.3\left(obs\right)}}}$$

where ${BT}_{10.5\left(calc\right)}$ is the *BT* calculated using the radiative transfer model; ${BT}_{10.5\left(obs\right)}$ is the GK2A satellite-observed *BT*; ${BTD}_{10.5-12.3\left(calc\right)}$ is the difference between the ${BT}_{10.5}$ and ${BT}_{12.3}$ using the radiative transfer model; and ${BTD}_{10.5-12.3\left(obs\right)}$ is the satellite-observed BTD. 

#### 2.2.2. Empirical Bias Correction Method

Once the source of the empirical bias correction in the GK-2A DAOD was recognized, an algorithm was applied to correct for it. In addition, to generate a 24-h continuous forecast, the DAOD concentration was corrected by comparing it with the DAOD of the visible channel. Specifically, the CDF were used to adjust the GK-2A satellite IR (10.5 μm) AOD in accordance with visible AOD. This method is particularly convenient for harmonizing and facilitating the information gained from difference sources [50]. That is, instead of converting the wavelength, DAOD is converted into an empirical bias correction method using the wavelength value. The visible AOD of the GK-2A satellite had an accuracy of RMSE = 0.21 with a bias = 0.08 in 2020; it is being serviced and used for daytime monitoring. It is meaningful to use accuracy to correct the IR AOD based on Equations (25) and (26):

(25) $$DAOD\;(0<DAOD<1.6)=a_{0}+a_{1}DAOD+a_{2}{DAOD}^{2}+a_{3}{DAOD}^{3}$$

(26) $$DAOD\;(1.6\leq DAOD<4)=a_{4}+a_{5}DAOD+a_{6}{DAOD}^{2}+a_{7}{DAOD}^{3}$$

where the regression coefficients $a_{0}$ = 0.083, $a_{1}$ = −0.171, $a_{2}$ = 1.174, $a_{3}$ = −0.348 (0 < DAOD < 1.6), $a_{4}$ = −0.977, $a_{5}$ = 2.505, $a_{6}$ = −0.615, and $a_{7}$ = 0.075 (1.6 ≤ DAOD < 4) are gained through the fitting procedure, and the new DAOD were computed and selectively applied.

## 3. Results and Discussion

### 3.1. Dust Detection Using Infrared Channels from GK-2A

#### 3.1.1. Comparisons of Dust Imagery Products during the Nighttime

**Figure 4:**
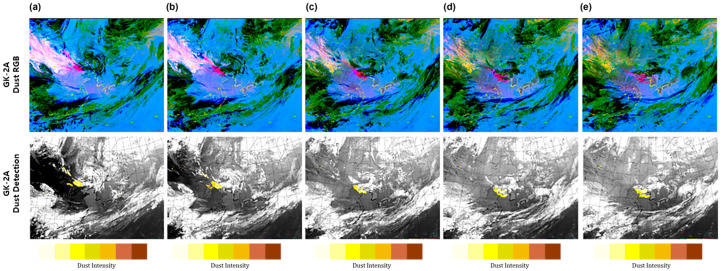
Example of the yellow dust detection using GK-2A’s infrared channel threshold values at (**a**) 07:00 UTC; (**b**) 10:00 UTC; (**c**) 13:00 UTC; (**d**) 16:00 UTC; and (**e**) 19:00 (24 May 2020, 03:00 UTC–11:00 UTC, 2-h intervals).

To monitor the DAOD for 24 h, accurately detecting dust at night using IR channels is essential. The dust red-green-blue (RGB) imagery from the GK-2A/AMI (developed by the KMA NMSC) was used for qualitative evaluation at nighttime. This imagery was developed by selecting an appropriate channel to detect dust and converting it to R (11.2–10.5 µm), G (10.5–8.7 µm), B (10.5–13.3 µm) using the BTD in the IR channel to monitor the occurrence and movement of dust. The images facilitate the visual discrimination between dust (purple-pink) and non-dust (green), enabling the identification of the cloud type. On 24 April 2020, at 03:00 UTC (12:00 KST), a dust storm originated east of Mongolia and northern China and moved to the southeastern regions due to northwesterly winds (Figure 4). A high concentration of 407 µm/m^3^ was observed in Huimin, which affected a weak intensity of <200 µm/m^3^ at 21:00 UTC (25 April 2020, at 05:00 KST). Subsequently, the storm began to affect the Yellow Sea at 22:00 UTC (25 April 2020, at 07:00 KST) and penetrated the Korean Peninsula at a concentration of 100–200 µm/m^3^ at 00:00 UTC (09:00 KST) on 25 April. As a result of the GK-2A aerosol detection product analysis, this image shows that dust originated, entered the Shandon Peninsula, and moved to the southwest well on 24 April 2020, at 07:00 UTC–10:00 UTC (Figure 4a,b). Additionally, based on the yellow dust well, the darker the color, the stronger the dust storm. At 13:00 UTC (22:00 KST), dust was detected in the Yellow Sea, which moved southwest with a constant intensity (Figure 4c). When dust approached the ocean, it was continuously detected without discontinuity between land and sea. In many cases, dust detection was not possible due to the weakening of the detection strength caused by contrasting surface properties. Moreover, when dust occurs in clouds, it is important to prevent dust removal by clouds to increase the accuracy of dust detection. Therefore, yellow dust was not removed by the clouds and was continuously detected from 16:00 UTC to 19:00 UTC (Figure 4d,e). GK-2A dust RGB showed a strong dust signal (pink) in northwest China and a weak dust signal (pale pink) in the vicinity of the Shandong Peninsula and the east sea of the Korean Peninsula. As a result of the qualitative comparison with GK-2A dust RGB, it appeared similar to the comparative GK-2A aerosol detection image, as it was detected using a strict threshold test and fewer pixels in the GK-2A dust RGB. 

#### 3.1.2. Comparison of Dust Imagery Products during the Daytime

**Figure 5:**
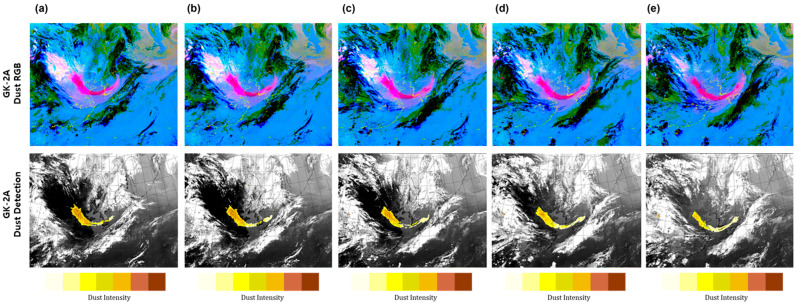
Example of the yellow dust detection using GK-2A’s infrared channel threshold values at (**a**) 03:00 UTC; (**b**) 05:00 UTC; (**c**) 07:00 UTC; (**d**) 09:00 UTC; and (**e**) 11:00 UTC (24 April 2020, 07:00 UTC–19:00 UTC, 3-h intervals).

For qualitative evaluation during the daytime, the dust RGB imagery was used, obtained through the same process described in Section 3.1.1. On 11 May 2020, at 03:00 UTC (12:00 KST), a dust storm originated in eastern Mongolia and extended to inner Mongolia, China (Figure 5). It was also observed on the Loess Plateau and west of the Shandong Peninsula at 00:00 UTC on 12 May (09:00 KST). According to the China Meteorological Administration (CMA), a high dust concentration of ≥800 µm/m^3^ was observed in inner Mongolia, such as in Erlenhot and Zhulihe, at 05:00 UTC on May 11, reaching a maximum of >3000 µm/m^3^. On 12 May, the concentration in Dungseong, located on the Loess Plateau, began to rise, indicating that it was approaching the Korean Peninsula as it was biased to the southwest. Subsequently, dust storms were detected in the Yellow Sea and the central area of the Korean Peninsula through the Shandong Peninsula in China. In particular, the dust across the Korean Peninsula was strong. As a result of GK-2A aerosol detection analysis, the image represented that dust was strongly penetrating the Korean Peninsula in a U-shape. From 03:00 UTC to 05:00 UTC on 12 May (Figure 5a,b), the concentration of dust generated strongly in southern China gradually weakened as it passed through the Yellow Sea, affecting the Korean Peninsula. Additionally, due to the improvement of the yellow dust detection algorithm, continuity between land and sea was well distinguished, enabling the identification of the dust flow. From 07:00 UTC to 09:00 UTC (Figure 5c,d), dust that had affected the central region of the Korean Peninsula moved to the south and directly affected the southern coast at 11:00 UTC (Figure 5e). By using the GK-2A dust RGB, detection of dust movement became possible. The dark pink pixels in the dust RGB and the pixels in the GK-2A aerosol detection algorithm coincided, facilitating the accurate identification of yellow dust.

### 3.2. Correction of DAOD Visible and Infrared Channels from GK-2A

#### 3.2.1. GK-2A Visible AOD Spatial Variability and Retrieval Accuracy

**Figure 6:**
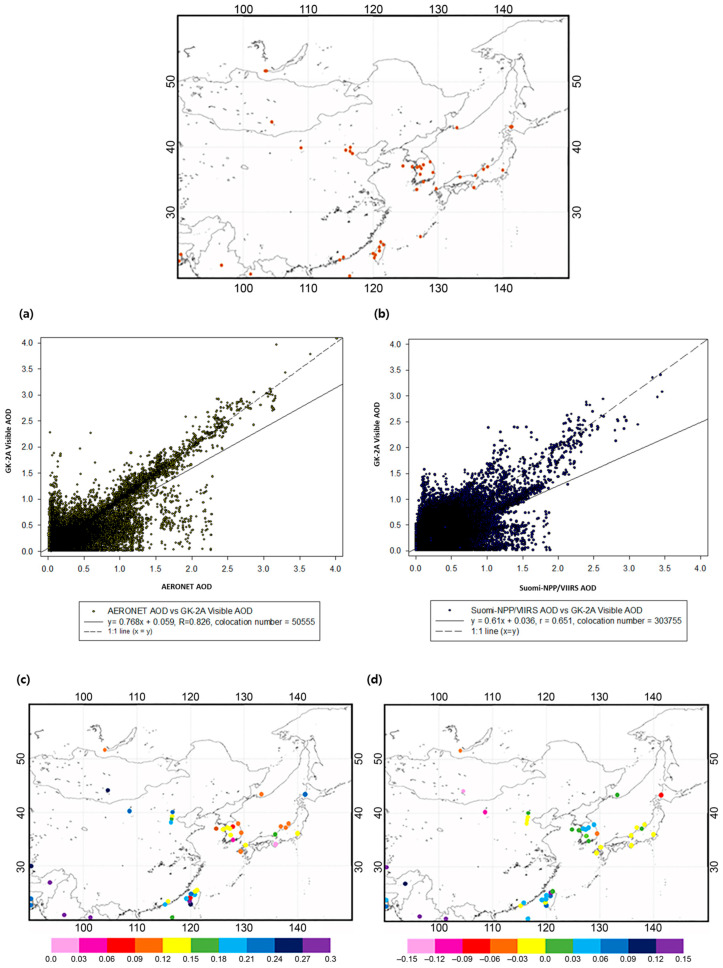
Locations of the 40 selected Aerosol Robotic Network (AERONET) sites used for comparing aerosol optical depth (AOD). Validation results of GK-2A AOD, AERONET (**a**), and Suomi-NPP/VIIRS (**b**) AOD in the 2020 yellow dust case. Results of statistical error (RMSE (**c**), bias (**d**)) analysis of GK-2A AOD and AERONET AOD.

Under the assumption that the accuracy of the visible AOD of GK-2A must be secured to a certain extent, CDF fitting can be performed on the IR AOD. Therefore, confirming the accuracy of the visible AOD was essential. To confirm the accuracy of the visible AOD, AERONET was used to provide ground observation data. The maps showing the location of the AERONET (Table 4) sun-sky radiometers utilized in this study are shown in Figure 6. AERONET data were used for cloud screening (Level 1.5) based on the dissimilar temporal frequencies between clouds and AOD. When the yellow dust occurred in East Asia in 2020, an AERONET point that matched the trajectory of the dust storm was selected, and only 40 sites could simultaneously utilize the data. The visible channel-based AOD and AERONET were compared for AERONET validation. Compared with AERONET AOD, GK-2A AOD values were near the 1:1 line, however, tended to be underestimated in some pixels. Figure 6a shows the R, RMSE, and bias values of 0.83, 0.23, and 0.084, respectively. Additionally, as the aerosol loading increased (AOD > 1), the values became increasingly close to the 1:1 line, indicating that the GK-2A AOD is superior in detecting high aerosol concentration plumes. However, the pixels with AERONET AOD were above 1.5 (AERONET AOD > 1.5); the low value of GK-2A AOD was due to the effect of aerosols being underestimated before calculating the GK-2A AOD by over-detecting the surface reflectivity of the 30-day background composite field. Additionally, to confirm the accuracy of the GK2A visible AOD, the aerosol products were validated using the Suomi-NPP/VIIRS (Figure 6b) EDR AOD in northeast Asia. The inter-comparison of GK-2A visible AOD products and VIIRS AOD indicated that the GK-2A visible AOD products had an overall global bias of −0.0008 against VIIRS EDR AOD, whereas the corresponding value of the VIIRS IP AOD products was0.0415 [13]. The validation of the GK-2A visible AOD revealed that the visible AOD retrievals agreed well with the VIIRS AOD observations (R = 0.651), and the GK-2A AOD products exhibited positive biases (*y* = 0.61*x* + 0.038) in a high aerosol loading area [51]. Similar to the AERONET results, when high-concentration aerosols were generated, the Suomi-NPP/VIIRS and GK-2A AOD were near the 1:1 line, showing agreement. However, when the concentration of VIIRS was high, the disadvantage of GK-2A under-detection appeared to be the same as that of AERONET, warranting further improvement in the future.

Focusing on these 40 AERONET sites, Figure 6c,d and Table 5 summarize the regional variations in GK-2A visible AOD retrieval accuracy. The GK-2A visible AOD performed better in Korea and Japan than in Taiwan and China. In particular, the GK-2A AOD had a lower RMSE and bias at points closer to the Korean Peninsula, while numerous errors occurred farther away. Moreover, the Korean Peninsula branching Anmyon, Hankuk-UFS, Seoul-University, Yonsei-University, Gwangju Gist, Socheongcho, and Gangneung-WNU had RMSEs as low as 0.07–0.13, and the bias (AERONET–Observation) was underestimated at 0.0003–0.05. However, in Japan, including Fukue, Fukuoka, Shirahama, Osaka, Chiba-University, Noto, Niigata, and Hokkaido, the RMSEs were as low as 0.07–0.24, and the bias was overestimated at 0.006–0.067, excluding Noto. Moreover, in China, including Xianghe, Beijing_CAMS, Beijing, and Beijing_radi, RMSEs of 0.146–0.20 and biases of −0.023 to 0.01 were detected. Hence, no overestimation occurred, similar to the Japanese region, but with a low RMSE. In contrast, the Taiwan region, including Dongsha_island, Luang_namtha, mandaly_mtu, Bhola, Hong Kong_polyU, Hong Kong_sheung, Kaoshiung, Chenkung_University, Lulin, Douliu, Dhaka_University, Xitun, EPA_NCU, Taipei_CWB, Fuguei_Cape, Dibrugarh_University, and NAM_CO, exhibited a relatively large RMSE and bias. Therefore, the accuracy of AERONET and GK-2A AOD by region tended to decrease as the distance increased from the Korean Peninsula. However, as this study focused on improving the accuracy of the IR and increasing the continuity of the DAOD during the day and night, it was conducted by considering the error of the AOD (assuming that the visible AOD is true).

#### 3.2.2. Bias Correction of GK-2A Infrared DAOD

**Figure 7:**
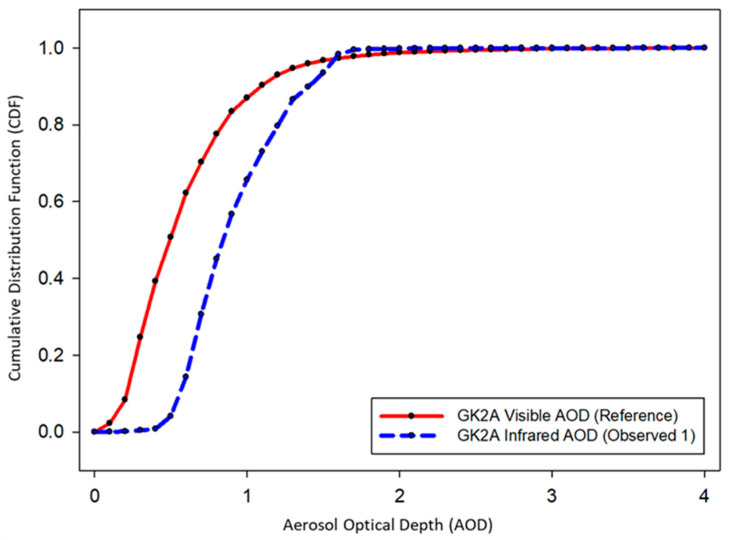
Cumulative distribution function (CDF) analysis results of visible and infrared aerosol optical depth (AOD) focusing on the 2020 yellow dust episodes.

**Figure 8:**
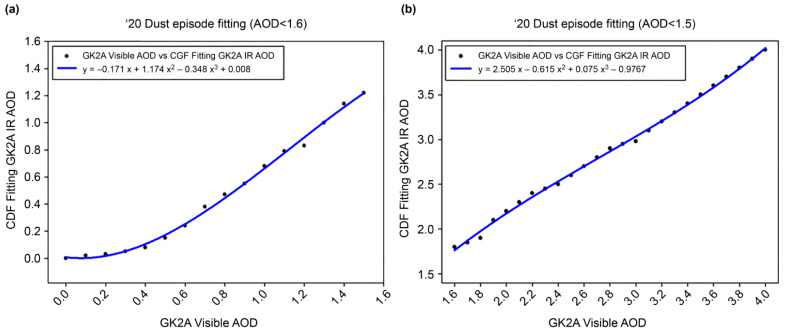
Cumulative distribution function (CDF) fitting analysis results of aerosol optical depth (AOD) < 1.6 (**a**) and AOD > 1.5 (**b**) focusing on the 2020 yellow dust episodes.

To maintain continuity in the visible and IR channels of the DAOD, CDFs were applied to the dust events in 2020. To validate the accuracy of DAOD, a representative case of yellow dust was tested using 2021 data. The goal was to maximize the accuracy of the DAOD value while tracking the accuracy of the visible-channel DAOD. This method assumes that the visible DAOD is true and applies a method for fitting the IR DAOD. That is, the visible DAOD is calculated based on reflectivity, and the IR DAOD is calculated based on radiation; hence, their calculation purposes differ, and each LUT differs, limiting consistency. However, the statistical CDF fitting method was adopted by focusing on the technology to correct errors quickly. CDFs were applied to individual grid cells to increase the accuracy of the IR DAOD based on the visible DAOD. The CDFs of DAOD during the training data (dust event in 2020) are shown in Figure 7. Notably, the visible DAOD was evenly distributed within the 0–4 range, while the IR DAOD = was cumulated in the 0.5–1.6 range. In addition, at the 1.6 starting point of the AOD, the visible and IR AOD crossed, and the fitting curve was reversed. That is, it did not detect low dust concentrations of 0–0.5 µm and tended to underdetect strong yellow dust. Since the DAOD of GK-2A was calculated only for yellow dust pixels, the sensitivity of DAOD using the IR channel was greater, and the concentration of DAOD tended to be high. In fact, because the DAOD of yellow dust was observed at a high concentration rather than at a low concentration of 0.5, this may result from reflection. Therefore, in this study, the tendency of the fitting curve differed based on a DAOD of 1.6; the coefficient was applied by bisecting it. The regression coefficients for each dust pixel were calculated; Figure 8 shows the piecewise linear CDF fitting across the GK-2A satellite imagers analyzed for 2020. From the CDF fitting curve, the IR DAOD was overestimated compared with the visible DAOD within the 0–1.6 range (Figure 8a), and the IR DAOD was underestimated compared to the visible DAOD within the 1.6–4.0 range (Figure 8b). Therefore, the coefficients were derived by dividing each DAOD concentration range. Although each range was set and applied, the frequency of the data was small in the DAOD range of 1.6 to 4.0, and the high concentration duration was short, indicating an even distribution of data. 

#### 3.2.3. Accuracy of Infrared DAOD Applied to Coefficient (Correction Factor)

**Figure 9:**
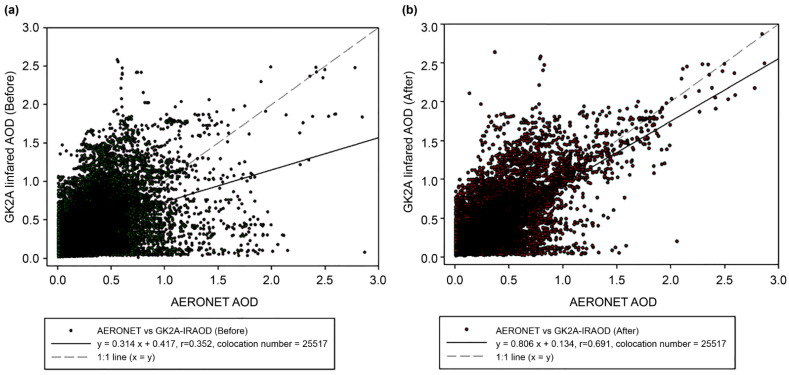
Validation results of GK-2A Infrared aerosol optical depth (AOD) before (**a**) and after (**b**) applying the cumulative distribution function (CDF) fitting method.

To validate accuracy, the IR DAOD data of the GK-2A was applied. Previous studies have applied the coefficients calculated using the CDF fitting method to the GK-2A DAOD. Meanwhile, the current study only collected data for dust detection pixels for the 2021 dust episodes. Figure 9 shows the scatterplots of AERONET versus GK-2A IR DAOD before (Figure 9a) and after (Figure 9b) bias correction for dust pixels. First, the DAOD before correction based on pixels co-located with AERONET showed very low accuracy based on the x = y (1:1) line. The original AMI DAOD had a correlation coefficient of 0.352, a slope of 0.314, and an offset of 0.417. AERONET was developed based on 500 nm (visible) and DAOD based on 1050 nm (IR); therefore, differences inevitably occurred. This study aimed to develop a nighttime DAOD and ensure continuous monitoring alongside daytime DAOD, which was challenging. However, the visible channel AOD and DAOD after CDF fitting showed improved results and a trend similar to the 1:1 line (after bias correction, the correlation improved to 0.691, and the slope and offsets improved to 0.806 and 0.134, respectively). It was estimated to be improved as the value of DAOD shows that over-detection is relieved when AERONET’s AOD (0.5–1.0) is small, and a similar tendency is shown when the concentration of the AERONET AOD is high. 

**Figure 10:**
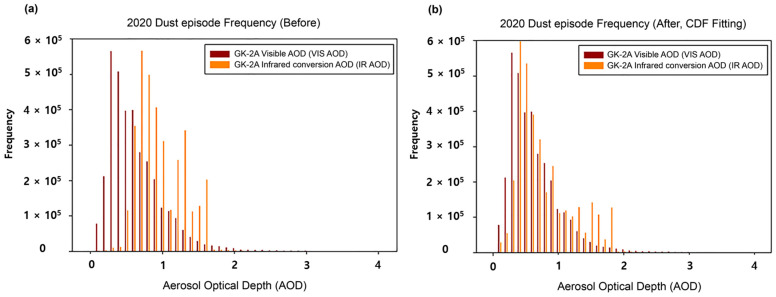
Histogram frequency results of GK-2A Infrared aerosol optical depth (AOD) before (**a**) and after (**b**) applying the cumulative distribution function (CDF) fitting method.

Figure 10 shows a histogram of the original (uncorrected, Figure 10a) and CDF fitting (corrected, Figure 10b) IR DAOD pixels over the dust detection pixels (the same observation time as the DAOD data shown in Figure 9). The standard visible AOD had a high frequency of ~0.3, gradually decreasing with increasing concentration. Although before bias correction, there were numerous IR DAOD frequencies in the range of 0.7, the detection was poor at concentrations < 0.5 and >1.6. This estimated DAOD only applies to pixels detected as dust, hence, when dust occurs, the concentration is high, and a small concentration < 0.5 cannot be detected even in the IR channel [52]. After applying the CDF method, the number showing high frequency at DAOD 0.7 shifted to the left, and the frequency showing low concentration increased. Therefore, the visible and IR DAOD were consistent, and their concentration distribution showed a similar tendency. 

### 3.3. Qualitative Comparisons through Intense Dust Events

Asian dust originates in the deserts of the Taklamakan, Mongolia, northern China, and Kazakhstan and travels to China, North and South Korea, and Japan, where it particularly impacts human health and air quality. [53,54]. It is a well-known phenomenon that occurs during springtime in northeast Asia and frequently in fall and winter. After applying the coefficients derived from the 2020 training dataset, the GK-2A DAOD was computed and applied for dust events that occurred in 2021.

#### 3.3.1. Spring Dust Transport over the Korean Peninsula on 23–28 March 2021

On 21 March 2021, yellow dust originated from the Gobi Desert and the inner Mongolian highlands. This dust was driven by a northwest wind on 23 March and was detected in northern and northeastern China and the Korean Peninsula. Meanwhile, the concentration of PM_10_ at 04:00 UTC in Sokcho was 262 µg/m^3^, in Daegwallyeong was 247 µg/m^3^, in Mungyeong was 206 µg/m^3^, and in Uljin was 162 µg/m^3^. The yellow dust that occurred on 21 March gradually faded, however was observed again on March 26 at 04:00 UTC in the inner Mongolian plateau, and the concentration of PM_10_ in Zuricher was 838 µg/m^3^ and in Erlenhot was 1226 µg/m^3^. Subsequently, PM_10_ was observed in certain areas of the Korean peninsula at 09:00 UTC with 141 µg/m^3^ in Seoul, 132 µg/m^3^ in Suwon, 105 µg/m^3^ in Ganghwa, 100 µg/m^3^ in Gwanaksan, and 100 µg/m^3^ in Gwangju. 

**Figure 11:**
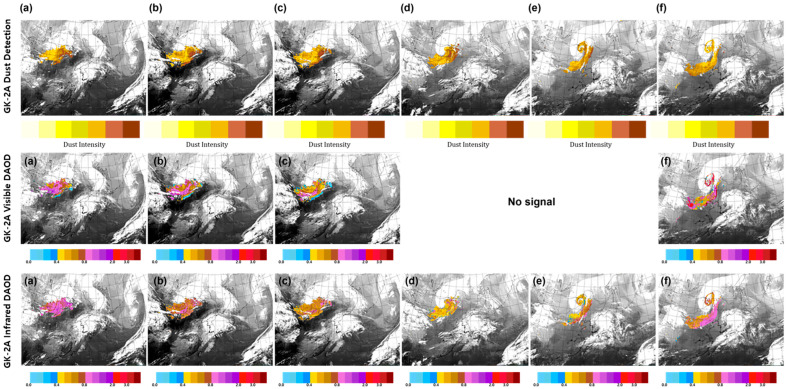
Images of Asian dust based on GK-2A Dust detection, GK-2A Visible Dust Aerosol Optical Depth (DAOD), GK-2A Infrared DAOD on 26–28 March 2021 (**a**) 26, 23:00 UTC; (**b**) 27, 04:00 UTC; (**c**) 27, 09:00 UTC; (**d**) 27, 14:00 UTC; (**e**) 27, 19:00 UTC; and (**f**) 28, 01:00 UTC.

Figure 11 shows the dust detection, visible DAOD, and IR DAOD obtained by GK-2A on 26–28 March from 23:00 UTC to 01:00 UTC at 5 h intervals based on the developed algorithm. Based on the improved results, continuity, and detection speed were increased regardless of the ground surface characteristics. Therefore, the area for detecting the DAOD at night increased, and the flow of yellow sand observed in Mongolia along the northwest wind could be effectively monitored. Over time, the yellow dust approached the Korean Peninsula, and when it originated, the DAOD of the visible and IR channels reached concentrations of ≥0.8 (Figure 11a). After 5 h, the concentration began to decrease to 0.4 (Figure 11b,c). Additionally, while the DAOD of the visible region was observed at various concentrations, the DAOD of the IR region exhibited a concentration of 0.4–0.8. After the twilight period, only the DAOD of IR could be observed (Figure 11d,e), confirming that the DAOD was observed at the same location as the dust detection, and clouds and dust were mixed while approaching the Korean Peninsula at 19:00 UTC. After 5 h (Figure 11f), visible and IR DAOD were observed during the daytime after dawn, and the concentration increased; however, the visible DAOD showed a higher concentration.

#### 3.3.2. Example of Dust Transport over the Korean Peninsula on 14–16 April 2021 (Spring Dust)

**Figure 12:**
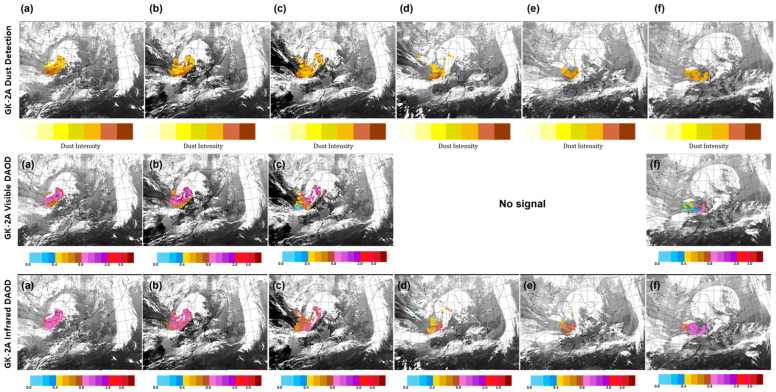
Images of Asian dust based on GK-2A Dust detection, GK-2A Visible Dust Aerosol Optical Depth (DAOD), GK-2A Infrared DAOD on 14–16 April 2021. (**a**) 14, 23:00 UTC; (**b**) 15, 04:00 UTC; (**c**) 15, 09:00 UTC; (**d**) 15, 14:00 UTC; (**e**) 15, 19:00 UTC; and (**f**) 16, 01:00 UTC.

On 14 April, yellow dust originating from the Gobi Desert affected Bohai Bay. At 04:00 UTC on 15 April, satellites detected dust in areas such as the Gobi Desert in southern Mongolia, northern China, and the Inner Mongolian Highlands. The concentrations of PM_10_ were 3342 µg/m^3^ at Zurcher, 1552 µg/m^3^ at Erlenhot, and 886 µg/m^3^ at Dongseong, which were affected by the high concentration of dust. Figure 12 shows the dust detection, visible DAOD, and IR DAOD obtained by GK-2A on 14–16 April, from 23:00 UTC to 01:00 UTC at 5 h intervals based on the use of the developed algorithm. A northeast–southwest directional V-shaped band passing through the West Sea first flowed in and affected the Korean Peninsula; as time passed, yellow dust was introduced again. When it first entered, a high concentration of ≥1.0 (Figure 12a–c) was observed approaching in the form of cloud penetration. Yellow sand mixed with clouds was detected, and its concentration decreased over time. At night (Figure 12d,e), dust was observed at the same location, and the concentration ranged from 0.4 to 1.0; the DAOD became thicker, penetrating the cloud. After dawn, the dust increased again during the day (Figure 12f), and both visible and IR DOAD appeared to be at the same concentration. 

#### 3.3.3. Example of Dust Transport over the Korean Peninsula on 4–8 May 2021 (Spring Dust)

**Figure 13:**
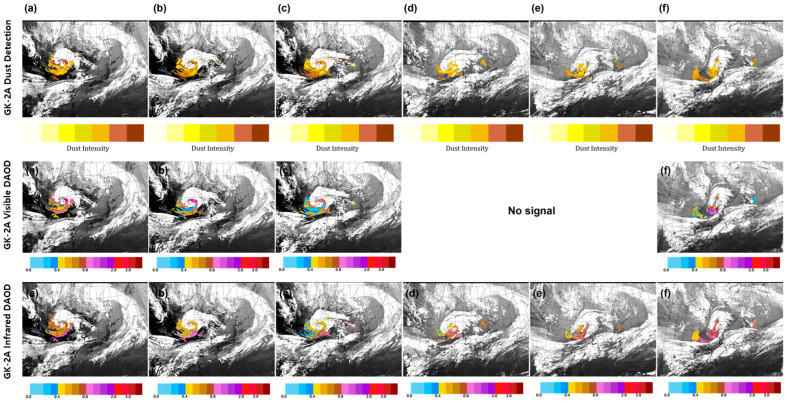
Images of Asian dust based on GK-2A Dust detection, GK-2A Visible Dust Aerosol Optical Depth (DAOD), GK-2A Infrared DAOD on 5–7 May 2021 (**a**) 5, 23:00 UTC; (**b**) 6, 04:00 UTC; (**c**) 6, 09:00 UTC; (**d**) 6, 14:00 UTC; (**e**) 6, 19:00 UTC; and (**f**) 7, 01:00 UTC.

The 5–8 May 2021, event was selected as the longest-lasting yellow dust event in 2021. On 3 May, the dust storm originated from the Gobi, Northern China, and Inner Mongolian Highlands and continued to affect the region until 7 May. Figure 13 shows the dust detection, visible DAOD, and IR DAOD obtained by GK-2A on 5–7 May, from 23:00 UTC to 01:00 UTC at 5 h intervals based on the use of the developed algorithm. The yellow dust piled up with clouds and approached the Korean Peninsula in a circle resembling a pig’s tail. When it originated in a form similar to the previous two cases, it showed a tendency to approach with a thick concentration and then fade; yellow dust appeared to penetrate between the cloud belt located under the Korean Peninsula and clouds located in China (Figure 13a–c) during the daytime. In particular, the DAOD values of 0.4 to 0.9 at night (Figure 13d,e) effectively monitored dust movement. Consequently, during the daytime, after 5 h (Figure 13f), the concentration of dust moving between clouds tended to be consistent with 1.0; however, in the flow of dust that followed, it appeared somewhat higher in the IR channel.

### 3.4. Quantitative Comparison between GK-2A DAOD and CALIPSO/CALIOP throughout Dust Events in 2021 (Validation)

**Figure 14:**
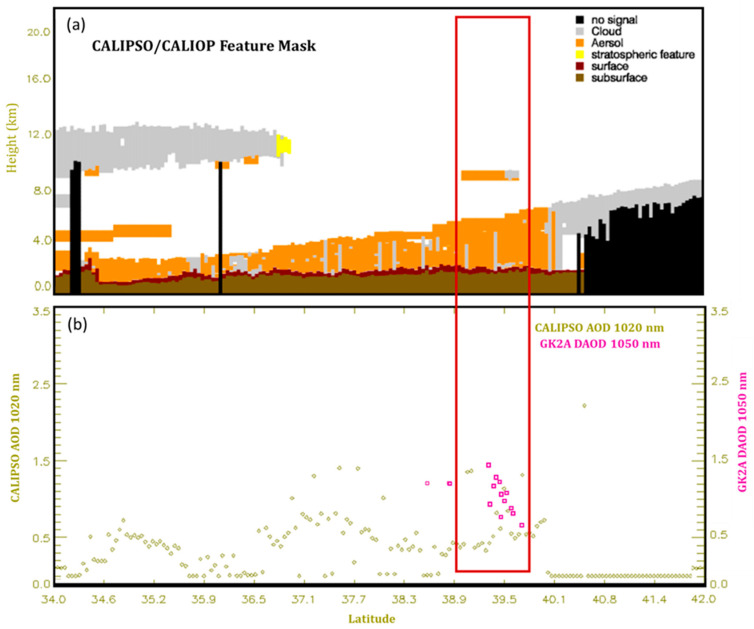
Validation results after matching CALIOP/CALIPSO Infrared aerosol optical depth (AOD) (1020 nm) and GK-2A Infrared AOD (1050 nm). (Red box means that pixels where GK-2A matches CALIPSO at night).

For the quantitative validation of the GK-2A DAOD, CALIPSO was used, which has a vertical profile in northeast Asia for 2021. Most of the associated DAOD was calculated only during the daytime and can, thus, be used to validate the DAOD calculated at night developed in this study. The CALIPSO satellite is a passive satellite that cannot detect large areas owing to its narrow swath. However, it was the only data to validate DAOD at night, and it is valuable data that can only be verified after passing that time and point. [55]. Since the CALIPSO satellite observes the Korean Peninsula once during the day and night, the time when the dust occurred and the time when the CALIPSO passed should be the same; therefore, there were limitations in the validation case. To validate the data, the CALIPSO/CALIOP AOD at 10.2 µm was used for retrieval during the 10-min observation period of the GK-2A IR DAOD. By selecting a point within a 10-km radius of the CALIPSO pixel, the GK-2A DAOD grid was determined to be the nearest point. The CALIPSO data and GK-2A matched the approach of yellow dust over the Korean Peninsula at 19:10 UTC on 15 April 2021 (CAL_LID_L2_VFM_ValStage1-V3-41.2021-04-15T-10-53ZN. hdf). Yellow dust was strongly detected in the GK-2A dust detection retrieval (Figure 14a), and GK-2A AOD (Figure 14b) was also observed at a high concentration (range: 0.7–1.3). In particular, within the CALIPSO observations at night, the yellow sand was found to be floating high at latitude 38° at the same location as GK-2A at approximately 8.5 to 9 km from CALIPSO 10.2 nm AOD, and the value was measured up to 1.3. In contrast, the low-lying yellow dust was estimated to be approximately 4 km, and the GK-2A DAOD and colocation pixels were the same at 0.7. When dust occurs at night, validation is difficult because the two satellites must match simultaneously. Nevertheless, meaningful results can be obtained by inferring the quantitative dust values, particularly at night, using the validation case. 

## 4. Summary and Conclusions

Based on the developed and improved IR DAOD of GK-2A, multispectral data can be obtained for dust plumes. In particular, based on the observation data analysis, we developed a dust storm mask algorithm to pinpoint dust detection, direction, and flow. The dust detection algorithm is based on the multi-threshold IR (8.7, 10.5, 11.2, and 12.3 µm) BTD-BBTD ratio. This algorithm can be used under 24-h conditions to identify yellow dust. In this study, the performance of the GK-2A visible AOD in northeast Asia was evaluated against AERONET and Suomi-NPP/VIIRS. The IR DAOD was presented utilizing a CDF fitting method to retrieve the column properties of atmospheric aerosols from the GK-2A visible AOD. The accuracy of GK-2A IR DAOD data was assessed using collocated measurements with ground observations, and spatial-temporal variability was examined using CALIPSO/CALIOP.

The main contributions of this study are as follows:

1. The GK-2A/AMI DAOD was developed to detect yellow sand in northeast Asia and achieve continuous monitoring during the day and night.
2. The GK-2A/AMI AOD had an overall high correlation with AERONET (R = 0.691) and Suomi-NPP/VIIRS (R = 0.651).
3. The developed DAOD exhibited variable accuracy in the visible AOD; thus, its accuracy was improved using a CDF fitting method, assuming that the visible AOD is true.
4. Following the DAOD correction, it was validated quantitatively and qualitatively with AERONET. In particular, when yellow dust appeared, the movement flow of the dust was monitored and showed continuity for 24 h.
5. Validation of the DAOD with CALIPSO/CALIOP at night showed quantitative values similar to 1020 nm for the CALIPO product, enabling the persistence of the height and attribute of the dust at the time of occurrence.

This study was performed to provide a method to calculate the DAOD even at night using the characteristics of the IR channel that can be applied for forecasting more accurate values. However, only dust pixels were used because yellow dust occurred, and among those pixels, there were few cases in which the time and space coincided with polar orbiting satellites; above all, verification was limited. In the future, the model will be applied to additional dust cases to perform further qualitative and quantitative validations and address lingering issues, including under-detection.

## Figures and Tables

**Table 1:** Summary of the GK-2A/AMI spectral bands.

Band	Band Name	Wavelength		Band Width (Max)	Spatial Resolution (km)
		Min (um)	Max (um)		
1 (blue)	VIS0.47	0.43	0.48	0.075	1
2 (green)	VIS0.51	0.52	0.52	0.063	1
3 (red)	VIS0.64	0.63	0.66	0.125	0.5
4 (VIS)	VIS0.86	0.85	0.87	0.088	1
5 (NIR)	NIR1.37	1.37	1.38	0.03	2
6 (NIR)	NIR1.61	1.60	1.62	0.075	2
7 (IR)	SWIR3.8	3.74	3.96	0.5	2
8 (IR)	WV6.3	6.06	6.43	1.038	2
9 (IR)	WV6.9	6.89	7.01	0.5	2
10 (IR)	WV7.3	7.26	7.43	0.688	2
11 (IR)	IR8.7	8.44	8.76	0.5	2
12 (IR)	IR9.6	9.54	9.72	0.475	2
13 (IR)	IR10.5	10.3	10.6	0.875	2
14 (IR)	IR11.2	11.1	11.3	1.0	2
15 (IR)	IR12.3	12.2	12.5	1.25	2
16 (IR)	IR13.3	13.2	13.4	0.75	2

**Table 2:** Dust events in 2020–2021 used this study.

Satellite Data Composition	Dust Event Date	Oriental Dust Location	Analysis Day
Training dataset dust episodes (2020)	16–17 February 2020	Dalian (Northeast of China)	00:00 UTC–10:50 UTC
	20–23 February 2020	Wulatezhongqi/Yanan~ Shandong province/ Bohai sea	
	12–14 March 2020	Tibetan Plateau~ Shandong province~ Bohai sea	
	18–19 March 2020	Inner Mongolia~Bohai sea	
	30 March–2 April 2020	Southeast of Mongolia	
	3–7 April 2020	Wulatezhongqi (East of Monolia)	
	15–18 April 2020	Mongolia	
	20–22 April 2020	Dandong~Bohai sea~ Tongliao, Siping	
	24–25 April 2020	East of Mongolia	
	10–11 May 2020	Bohia sea~Siping	
	11–14 May 2020	Inner Mongolia	
	31 May–6 June 2020	Gobi Desert~ Wulatezhongqi~ Erenhot~Jurihe	
	5–11 June 2020	Taklamakan	
	9–11 June 2020	Gobi Desert	
	19–23 October 2020	Gobi Desert	
	30 October–2 November 2020	Gobi Desert	
	5–8 November 2020	Gobi Desert	
	7–8 November 2020	Manchuria	
Analysis dataset dust edpisodes (2021)	13–14 January 2021	Gobi Desert	00:00 UTC–23:50 UTC
	21–22 February 2021	Gobi Desert	
	15–17 March 2021	Mongolia	
	23–28 March 2021	Gobi Desert	
	15–17 April 2021	Mongolia	
	26–27 April 2021	Mongolia	
	04–07 May 2021	Gobi Desert	
	23–24 May 2021	Gobi Desert	
	16 December 2021	Inner Mongolia	

**Table 3:** List of the input variables used to calculate the dust aerosol optical depth (DAOD) lookup table.

Variable Name	Number of Entries	Entries
Wavelength	5	3.8, 10.5, 11.2, 12.4, 13.3 μm (considering spectral response function)
Solar zenith angle	9	0, 10, 20, 30,..., 80 (10 intervals)
Satellite zenith angle	17	0, 5, 10, 15,..., 80 (5 intervals)
Relative azimuth angle	18	0, 10, 20,..., 170 (10 intervals)
AOD	10	0.0, 0.3, 0.6, 0.9, 1.2, 1.5, 2.0, 3.0, 4.0, 5.0
Dust Aerosol model	10	1, 2, 3, 4, 5, 6, 7, 8, 9, 10 μm (considering effective radius)
Dust Altitude	10	1, 2, 3, 4, 5, 6, 7, 8, 9, 10 km

**Table 4:** Details about the representative Aerosol Robotic Network (AERONET) site to validate dust aerosol optical depth (DAOD).

Site	Latitude (Degree)	Longitude (Degree)	Elevation (m)	Type
Anmyon	36.539 N	126.330 E	47	Rural
AOE_Baotou	40.852 N	109.629 E	1314	Rural
Beijing	39.977 N	116.381 E	92	Urban
Beijing-CAMS	39.933 N	116.317 E	106	Urban
Beijing-RADI	40.005 N	116.379 E	59	Urban
Bhola	22.227 N	90.756 E	7	
Chen-Kung_Univ	22.993 N	120.204 E	50	
Chiba_University	35.625 N	140.104 E	60	
Dalanzadgad	43.577 N	104.419 E	1470	Rural
Dhaka_University	23.728 N	90.398 E	34	
Dibrugarh_Univ.	27.451 N	94.896 E	119	
Dongsha_island	20.699 N	116.729 E	5	
Douliu	23.712 N	120.545 E	60	
EPA-NCU	24.968 N	121.185 E	144	
Fuguei_Cape	25.297 N	121.538 E	50	
Fukue	32.752 N	128.682 E	80	
Fukuoka	33.524 N	130.475 E	30	
Gangneung_WNU	37.771 N	128.867 E	60	Suburban
Gwangju_GIST	35.228 N	126.843 E	52	Urban
Hankuk_UFS	37.339 N	127.266 E	167	
Hokkaido_University	43.075 N	141.341 E	59	
Hong_Kong_PolyU	22.393 N	114.180 E	30	
Hong_Kong_Sheung	22.483 N	114.117 E	40	
Irkutsk	51.800 N	103.087 E	670	
Kaohsiung	22.676 N	120.292 E	15	
Luang_Namtha	20.931 N	101.416 E	557	
Lulin	23.469 N	120.874 E	2868	
Mandalay_MTU	21.973 N	96.186 E	104	
NAM_CO	30.773 N	90.963 E	4746	
Niigata	37.846 N	138.942 E	10	
Noto	37.334 N	137.137 E	200	
Osaka	34.651 N	135.591 E	50	
Seoul_SNU	37.458 N	126.951 E	116	Urban
Shirahama	33.693 N	135.357 E	10	
Socheongcho	37.423 N	124.738 E	28	Ocean
Taipei_CWB	25.015 N	121.538 E	26	
Ussuriysk	43.700 N	132.163 E	280	
XiangHe	39.754 N	116.962 E	36	Urban
Xitun	24.162 N	120.617 E	91	
Yonsei_University	37.564 N	126.935 E	97	Urban

**Table 5:** Results of statistical error (RMSE, bias) analysis for the representative Aerosol Robotic Network (AERONET) site to validate GK-2A of Aerosol Optical Depth (AOD) results.

Site	Latitude/Longitude (Degree)	Colocation Number	RMSE	Bias
Anmyon	36.539 N/126.330 E	1675	0.122	0.009
AOE_Baotou	40.852 N/109.629 E	1156	0.225	−0.111
Beijing	39.977 N/116.381 E	1031	0.146	−0.023
Beijing-CAMS	39.933 N/116.317 E	1975	0.177	−0.017
Beijing-RADI	40.005 N/116.379 E	1871	0.191	−0.02
Bhola	22.227 N/90.756 E	1871	0.211	0.047
Chen-Kung_Univ	22.993 N/120.204 E	1404	0.273	0.062
Chiba_University	35.625 N/140.104 E	1165	0.13	−0.023
Dalanzadgad	43.577 N/104.419 E	2133	0.264	−0.155
Dhaka_University	23.728 N/90.398 E	681	0.224	0.078
Dibrugarh_Univ.	27.451 N/94.896 E	948	0.279	0.101
Dongsha_island	20.699 N/116.729 E	1526	0.176	0.052
Douliu	23.712 N/120.545 E	722	0.185	0.029
EPA-NCU	24.968 N/121.185 E	994	0.193	0.024
Fuguei_Cape	25.297 N/121.538 E	604	0.134	0.016
Fukue	32.752 N/128.682 E	1689	0.095	−0.006
Fukuoka	33.524 N/130.475 E	1767	0.141	−0.008
Gangneung_WNU	37.771 N/128.867 E	1343	0.13	0.032
Gwangju_GIST	35.228 N/126.843 E	565	0.134	0.003
Hankuk_UFS	37.339 N/127.266 E	1800	0.073	0.055
Hokkaido_University	43.075 N/141.341 E	918	0.237	−0.067
Hong_Kong_PolyU	22.393 N/114.180 E	591	0.18	−0.006
Hong_Kong_Sheung	22.483 N/114.117 E	860	0.13	0.006
Irkutsk	51.800 N/103.087 E	1105	0.101	−0.049
Kaohsiung	22.676 N/120.292 E	1626	0.24	0.062
Luang_Namtha	20.931 N/101.416 E	1745	0.496	0.222
Lulin	23.469 N/120.874 E	630	0.09	−0.013
Mandalay_MTU	21.973 N/96.186 E	2299	0.303	0.154
NAM_CO	30.773 N/90.963 E	639	0.261	−0.139
Niigata	37.846 N/138.942 E	1384	0.106	−0.014
Noto	37.334 N/137.137 E	1329	0.12	0.014
Osaka	34.651 N/135.591 E	1371	0.182	−0.023
Seoul_SNU	37.458 N/126.951 E	1655	0.137	0.034
Shirahama	33.693 N/135.357 E	516	0.028	−0.006
Socheongcho	37.423 N/124.738 E	1000	0.115	0.008
Taipei_CWB	25.015 N/121.538 E	761	0.248	0.126
Ussuriysk	43.700 N/132.163 E	870	0.094	0.012
XiangHe	39.754 N/116.962 E	1388	0.202	0.002
Xitun	24.162 N/120.617 E	1486	0.233	0.053
Yonsei_University	37.564 N/126.935 E	1436	0.136	0.049

## Data Availability

Data are available on request owing to restrictions such as privacy or ethics. Data presented in this study are available upon request from the corresponding author.
